# Antioxidant Protective Effect of Glibenclamide and Metformin in Combination with Honey in Pancreas of Streptozotocin-Induced Diabetic Rats

**DOI:** 10.3390/ijms11052056

**Published:** 2010-05-05

**Authors:** Omotayo Owomofoyon Erejuwa, Siti Amrah Sulaiman, Mohd Suhaimi Abdul Wahab, Sirajudeen Kuttulebbai Nainamohammed Salam, Md Salzihan Md Salleh, Sunil Gurtu

**Affiliations:** 1 Department of Pharmacology, School of Medical Sciences, Universiti Sains Malaysia, 16150 Kubang Kerian, Kelantan, Malaysia; E-Mails: sbsamrah@kb.usm.my (S.A.S.); msuhaimi@kb.usm.my (M.S.A.W.); 2 Department of Chemical Pathology, School of Medical Sciences, Universiti Sains Malaysia, 16150 Kubang Kerian, Kelantan, Malaysia; E-Mail: sirajuden@kb.usm.my; 3 Department of Pathology, School of Medical Sciences, Universiti Sains Malaysia, 16150 Kubang Kerian, Kelantan, Malaysia; E-Mail: matledpb@yahoo.com; 4 School of Medicine and Health Sciences, Monash University Sunway Campus, Jalan Lagoon Selatan, 46150, Bandar Sunway, Selangor, Malaysia; E-Mail: sgurtu@gmail.com

**Keywords:** diabetes mellitus, streptozotocin, oxidative stress, pancreas, tualang honey, glibenclamide, metformin

## Abstract

Hyperglycemia exerts toxic effects on the pancreatic β-cells. This study investigated the hypothesis that the common antidiabetic drugs glibenclamide and metformin, in combination with tualang honey, offer additional protection for the pancreas of streptozotocin (STZ)-induced diabetic rats against oxidative stress and damage. Diabetes was induced in male Sprague Dawley rats by a single dose of STZ (60 mg/kg; ip). Diabetic rats had significantly elevated levels of lipid peroxidation (TBARS), up-regulated activities of superoxide dismutase (SOD) and glutathione peroxidase (GPx) while catalase (CAT) activity was significantly reduced. Glibenclamide and metformin produced no significant effects on TBARS and antioxidant enzymes except GPx in diabetic rats. In contrast, the combination of glibenclamide, metformin and honey significantly up-regulated CAT activity and down-regulated GPx activity while TBARS levels were significantly reduced. These findings suggest that tualang honey potentiates the effect of glibenclamide and metformin to protect diabetic rat pancreas against oxidative stress and damage.

## 1. Introduction

Diabetes mellitus is associated with progressive metabolic derangement, worsening glycemic control and morphological changes in the kidney, retina, pancreas and other organs [1–3]. Oxidative stress is known to play a significant role in the induction of these processes [4,5]. High levels of oxidative stress with excessive generation of free radicals and depleted levels of free radical scavenging enzymes have been demonstrated in several studies, both in experimental animal models of diabetes and in human diabetic subjects [6–8]. In type 1 diabetes, reactive oxygen species (ROS) are involved in *β*-cell dysfunction initiated by autoimmune reactions and inflammatory cytokines [9]. In type 2 diabetes, ROS activate β cell apoptotic pathways, impair insulin synthesis and also contribute to insulin resistance [10,11]. While oral hypoglycemic agents may be effective for glycemic control, at least in the early stages of diabetes, they do not appear to be effective in entirely preventing the progression of ROS mediated organ damage [3,12,13].

Tualang honey has previously been reported to attenuate free radical scavenging enzymes and reduce lipid peroxidation in kidneys of streptozotocin (STZ)-induced diabetic rats [14]. In this study, we examine the effect of the two most commonly prescribed antidiabetic drugs, glibenclamide and metformin, as well as tualang honey on oxidative stress in the pancreas of diabetic rats.

## 2. Methods

Thirty-six male Sprague-Dawley rats aged 10–12 weeks (250–300 g) from the Laboratory Animal Research Unit were used in this study. The rats were housed at 25 ± 2 °C under 12 hour cycles of dark and light and were allowed standard food and water *ad libitum.*

The rats were fasted for 18 hours before induction of diabetes. Diabetes was induced by a single intraperitoneal injection of freshly prepared streptozotocin (60 mg/kg) dissolved in 0.1 M citrate buffer (pH 4.5). Another group of rats (control) were injected with the same volume of citrate buffer. Streptozotocin (STZ)-injected rats exhibited symptoms of diabetes mellitus such as polyuria, polydipsia, polyphagia, and weight loss after 48 hours post STZ administration. Two days after the injection of STZ, fasting blood glucose concentration was measured with an Accu-Chek glucometer (Roche, Germany) using blood samples from the tip of the tail. Animals with blood glucose concentrations ≥ 14 mmol/L were considered diabetic and used in this study. Subsequently, fasting blood glucose was measured weekly in each rat. The rats were divided into 4 groups of 6 rats each. Using oral gavage, once each morning the rats were treated with tualang honey (AgroMas®, Malaysia), glibenclamide and/or metformin for four weeks (see Table 1). At the end of 4 weeks, the animals were sacrificed and pancreatic tissue excised and processed. The Universiti Sains Malaysia Animal Ethics Committee approved this study.

### 2.1. Processing and Preparation of Tissue

The pancreata were rapidly excised, washed in ice-cold normal saline, blotted, frozen in liquid nitrogen, and stored at −80 °C until use. 10% (w/v) homogenation of pancreatic tissues were made in Tris-HCl (0.1M, pH 7.4) using an ice-chilled glass homogenizing vessel in a homogenizer fitted with Teflon pestle (Glas-Col, USA) at 900 rpm. The suspended mixture was centrifuged at 1000 × g for 10 min at 4 °C in a refrigerated centrifuge. The resulting supernatant was used for the assay of activities of antioxidant enzymes, malondialdehyde concentrations and total protein.

### 2.2. Superoxide Dismutase (SOD) Assay

Superoxide dismutase (SOD) activity was measured using assay kit (Cayman, MI, USA) according to manufacturer’s instructions. This kit utilizes a tetrazolium salt for the detection of superoxide radicals generated by xanthine oxidase and hypoxanthine. One unit of SOD was defined as the amount of enzyme needed to produce 50% dismutation of superoxide radical. The SOD assay measures all the three types of SOD (Cu/Zn, Mn, and FeSOD).

### 2.3. Glutathione Peroxidase (GPx) Assay

Glutathione peroxidase (GPx) activity was measured using assay kit (Cayman, MI, USA) according to manufacturer’s instructions. The measurement of GPx activity is based on the principle of a coupled reaction with glutathione reductase (GR). The oxidized glutathione (GSSG) formed after reduction of hydroperoxide by GPx is recycled to its reduced state by GR in the presence of NADPH. The oxidation of NADPH is accompanied by a decrease in absorbance at 340 nm. One unit of GPx was defined as the amount of enzyme that catalyzes the oxidation of 1 nmol of NADPH per minute at 25 °C.

### 2.4. Catalase (CAT) Assay

CAT activity was assayed according to the method of Goth [15]. In brief, this assay involves the incubation of a test tube containing 0.5 mL of hydrogen peroxide and 0.1 mL of pancreatic homogenate. After incubation in a water bath at 37 °C for 60 seconds, the reaction was terminated by adding 0.5 mL of ammonium molybdate solution. A yellow complex of ammonium molybdate and hydrogen peroxide was formed. The absorbance of this yellow color was measured at 405 nm using spectrophotometer. One unit of CAT was defined as the amount of enzyme that catalyzes the decomposition of 1 μmol of hydrogen peroxide per minute.

### 2.5. Glutathione Reductase (GR) Assay

Glutathione reductase (GR) activity was measured according to the procedure of Goldberg and Spooner using oxidized glutathione (GSSG) as a substrate [16]. Briefly, 1 mL of 2.728 Mm GSSG solution and 40 μL of pancreatic homogenate were incubated in a water bath at 37 °C. After incubation for 5 minutes, the reaction was initiated by addition of 200 μL of 1.054 mM NADPH solution. The decrease in absorbance was measured at 340 nm using spectrophotometer and recorded every 30 seconds over a period of 5 minutes. GR activity was expressed as unit per mg protein based on molar extinction coefficient of 6.22 × 10^3^ L/mol/cm. One unit of GR was defined as the amount of enzyme that catalyzes the oxidation of 1 nmol of NADPH per minute.

### 2.6. Glutathione-S-Transferase (GST) Assay

Glutathione-S-transferase (GST) activity was assayed according to the method of Habig *et al* [17]. This procedure is based on the conjugation of glutathione (GSH) to 1-chloro-2,4-dinitrobenzene (CDNB) as a substrate. Briefly, 2 mL of 0.3 M potassium phosphate buffer (pH 6.35), 75 μL of 30mM CDNB solution, 725 μL of distilled water and 0.1 mL of pancreatic homogenate were pipetted into a test tube. The test tube was vortexed and incubated at 37 °C for 10 minutes. After incubation, the reaction was initiated by addition of 100 μL of 30 mM reduced glutathione solution. The decrease in absorbance was measured spectrophotometrically at 340 nm and recorded every 30 seconds for 4 minutes. GST activity was calculated as unit per mg protein based on a molar extinction coefficient of 9.6 × 10^3^ L/mol/cm. One unit of GST was defined as the amount of enzyme that catalyzes the conjugation of 1 nmol of GSH-CDNB per minute.

### 2.7. Lipid Peroxidation (LPO) Assay

The extent of lipid peroxidation was determined as the concentration of thiobarbituric acid reactive substances (TBARS), accordin*g* to the method of Ohkawa *et al*. [18]. Briefly, 100 μL of pancreatic homogenates or MDA standards were pipetted into test tubes containing 1.5 mL of 20% (w/v) glacial acetic acid (pH 3.5), 200 μL of 8.1% (w/v) sodium dodecyl sulphate (SDS), 1.5 mL of 0.8% (w/v) thiobarbituric acid (TBA) and 700 μL of distilled water. The test tubes were incubated at 95 °C for 60 minutes with a marble on top of each test tube. After incubation, the test tubes were cooled and then centrifuged at 3000 × g for 10 minutes. The amount of malondialdehyde (MDA) formed was measured spectrophotometrically at 532 nm. 1,1,3,3-Tetraethoxypropane (TEP), a form of MDA, was used as standard in this assay. TBARS concentration was expressed as nmol of malondialdehyde (MDA) per mg protein.

### 2.8. Protein Assay

Protein concentration was estimated using Bio-Rad protein assay kit based on the method of Bradford [19]. A standard curve was generated using bovine serum albumin as the standard.

### 2.9. Statistical Analysis

Statistical analysis was carried out using SPSS 12.0.1. The data were expressed as medians (interquartile range). Groups were compared by the Kruskal-Wallis H test followed by Mann-Whitney *U* test to identify significance of difference between two groups. *P* < 0.05 was considered to be statistically significant while n = 6.

## 3. Results

### 3.1. Lipid Peroxidation

**Figure 1.**
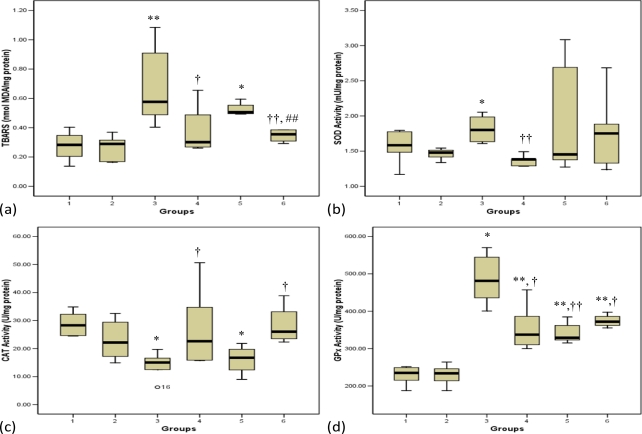
Effects of honey, glibenclamide and metformin as well as their combination on **(a)** concentrations of thiobarbituric acid reactive substances (TBARS), **(b)** activity of superoxide dismutase (SOD), **(c)** activity of catalase (CAT) and **(d)** activity of glutathione peroxidase (GPx) in pancreas of control and streptozotocin-induced diabetic rats. Group 1 (Normal control); Group 2 (Normal + Honey); Group 3 (Diabetic control); Group 4 (Diabetic + Honey); Group 5 (Diabetic + Glibenclamide + Metformin); Group 6 (Diabetic + Glibenclamide + Metformin + Honey). Each group consisted of five to seven animals. Data are expressed as median (interquartile range). Values are statistically significant at *p < 0.05, **p < 0.01 compared with group 1; †p < 0.05, ††p < 0.01 compared with group 3 and ## p< 0.01 compared with group 5. One unit of SOD was defined as the amount of enzyme needed to exhibit 50% dismutation of superoxide radical. One unit of CAT was defined as the amount of enzyme that catalyzes the decomposition of 1 μmol of H_2_O_2_ per minute. One unit of GPx was defined as the amount of enzyme that catalyzes the oxidation of 1 nmol of NADPH per minute.

Figure 1a depicts the concentrations of thiobarbituric acid reactive substances (TBARS) in normal and STZ-treated pancreas. The diabetic control rats showed significantly (p < 0.01) elevated levels of TBARS compared with normal control rats. The diabetic groups that received honey or a combination of glibenclamide, metformin and honey showed a significant (p < 0.05, group 4; p < 0.01, group 6, respectively) decrease in TBARS levels compared with the diabetic control group. On the contrary, administration of glibenclamide and metformin to diabetic rats did not produce any significant difference on TBARS levels compared with diabetic control rats (Figure 1a). Moreover, the diabetic rats that received a combination of glibenclamide, metformin and honey showed a significant (p < 0.01) decrease in TBARS levels compared with those administered glibenclamide and metformin.

### 3.2. Antioxidant Enzymes

The activities of glutathione reductase (GR) and glutathione-S-transferase (GST) in the pancreas of normal and STZ-treated rats did not change (results not shown). Superoxide dismutase (SOD) activity was significantly (p < 0.05) elevated in the diabetic control group (group 3) compared with normal control rats (group 1) (Figure 1b). The honey-treated diabetic rats (group 4) showed significantly (p < 0.05) reduced SOD activity, whereas neither glibenclamide and metformin nor their combination reduced SOD activity in diabetic rats. Catalase (CAT) activity in the pancreas was significantly (p < 0.05) reduced in the diabetic control group 3 compared with the group 1 control rats (Figure 1c). The diabetic rats that received glibenclamide and metformin showed reduced catalase activity similar to the diabetic control group. Administration of honey or a combination of glibenclamide, metformin and honey significantly (p < 0.05) up-regulated CAT activity towards the normal control group (Figure 1c). The diabetic control rats exhibited up-regulated activity of glutathione peroxidase (GPx) compared to normal control rats. Administration of honey, glibenclamide and metformin or their combination to diabetic rats significantly (p < 0.05, groups 4 and 6; p < 0.01, group 5) reduced GPx activity (Figure 1d).

### 3.3. Blood Glucose

**Figure 2.**
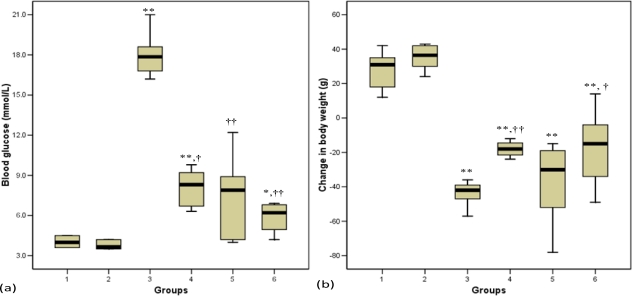
Effects of honey, glibenclamide and metformin as well as their combination on **(a)** blood glucose concentrations and **(b)** change in body weight of control and streptozotocin-induced diabetic rats. Each group consisted of five to seven animals. Group 1 (Normal control); Group 2 (Normal + Honey); Group 3 (Diabetic control); Group 4 (Diabetic + Honey); Group 5 (Diabetic + Glibenciamide + Metformin); Group 6 (Diabetic + Glibenciamide + Metformin + Honey). Data are expressed as median (interquartile range). Values are statistically significant at * p < 0.05, ** p < 0.01 compared with group 1 and †p < 0.05, ††p < 0.01 compared with group 3. Change in body weight is the difference between initial body weight before treatment and final body weight at the end of the treatment period.

Figure 2a shows the levels of blood glucose at the end of the experimental period. Before treatment commenced, the diabetic control rats (group 3) and diabetic-treated groups (groups 4, 5 and 6) had comparable levels of blood glucose (results not shown). The diabetic rats had significantly (p < 0.01) elevated blood glucose concentrations compared with non-diabetic rats. After four weeks of treatment, all the diabetic rats that received honey, glibenclamide and metformin or their combination had significantly (p < 0.05, group 4; p < 0.01, groups 5 and 6) reduced blood glucose concentrations compared with diabetic control rats (Figure 2a).

### 3.4. Body Weight

Figure 2b shows the change in body weight at the end of the experimental period. Before the commencement of this study, all the groups had similar body weight (data not shown). The body weight was significantly (p < 0.01) reduced in STZ-treated rats compared with normal control rats (group 1). The glibenclamide and metformin treated diabetic rats did not show significant weight gain. However, the diabetic groups that received honey or glibenclamide and metformin in combination with honey exhibited significant (p < 0.01, group 4; p < 0.05, group 6) improvement in weight gain compared with diabetic control rats (Figure 2b).

## 4. Discussion

The role of oxidative stress in the pathogenesis and complications of diabetes mellitus is well recognized [4–5,11]. Both human and experimental animal models of diabetes exhibit high oxidative stress due to persistent and chronic hyperglycemia, which depletes the activity of free radical scavenging enzymes and thus promotes free radicals generation [6–8]. Oxidative stress has lately been reported to be responsible, to a certain extent, for the β-cell dysfunction caused by glucose toxicity [20].

The pancreatic β-cells are highly prone to oxidative stress and damage because they have low expression and activity of antioxidant enzymes, which are the first line of defense against oxidative insult [21]. In this study, we found that the activity of superoxide dismutase (SOD) was up-regulated in the diabetic pancreas. SOD catalyzes the dismutation of superoxide (O_2_^•−^) radical to hydrogen peroxide (H_2_O_2_) [22]. Although SOD is an antioxidant enzyme, some studies have suggested that its overexpression is actually harmful to cells [23]. The toxic effect of ROS observed in many cells with overexpressed SOD has been linked to elevated levels of H_2_O_2_ and accompanying oxidative damage following hydroxyl radical formation [24]. The implication for SOD up-regulation is that there would be high turnover of H_2_O_2_. Since CAT, which inactivates H_2_O_2_ is an endogenous enzyme and needs to be replenished; the continuous formation of H_2_O_2_ might overwhelm this enzyme. Moreover, O_2_^•−^ is reported to inhibit CAT directly [25] so the ROS could have caused its reduced activity in the diabetic rats.

Glutathione peroxidase (GPx) is reported to have a broader protective spectrum than CAT because in addition to H_2_O_2_, it also metabolizes other hydroperoxides including lipid hydroperoxides [26]. The accumulation of H_2_O_2_ and other hydroperoxides might have induced the activity of GPx leading to its up-regulation in the diabetic rats. The observation that rodent and human islets have reduced expression of GPx has led to the conclusion that a normal approach for protection of β-cells against oxidative stress would involve over-expression of GPx [27]. Thus, the over-expression of GPx could be a protective mechanism against oxidative stress. Administration of honey up-regulated CAT activity and down-regulated the activity of SOD and GPx, thus indicating that honey attenuated the changes in the pancreatic antioxidant enzymes in response to generation of oxidants. On the other hand, glibenclamide and metformin down-regulated activity of GPx but did not produce any effect on CAT activity. However, the diabetic rats that received glibenclamide and metformin in combination with honey showed over-expressed CAT activity and also down-regulated activity of GPx indicating that glibenclamide and metformin in combination with tualang honey provide better attenuation of antioxidant enzymes.

Lipid peroxidation of unsaturated fatty acids is a frequently used indicator of increased oxidative stress and subsequent oxidative damage [28]. The enhanced activity of SOD and reduced CAT activity might generate excessive H_2_O_2_, which could give rise to other ROS such as hydroxyl radicals [23,24]. Lipid peroxidation impairs cell membrane fluidity and alters the activity of membrane-bound enzymes and receptors resulting in membrane malfunction [29]. The high level of lipid peroxidation marker, TBARS, in the diabetic control rats is a reflection of insufficiency of antioxidant defenses in combating ROS-mediated damage. This further shows that the up-regulation of SOD and GPx activities in the diabetic rats was an adaptive/defense mechanism but not protective against oxidative stress. The diabetic rats that received glibenclamide and metformin alone had significantly higher levels of TBARS than those that received glibenclamide and metformin in combination with tualang honey. This seems to suggest that glibenclamide and metformin does not offer protection against lipid peroxidative damage in pancreas. Therefore, this implies that honey offers additional antioxidant effect to glibenclamide and metformin, thereby protecting the pancreas from oxidative stress-induced damage.

In STZ-induced diabetic rats, diabetes develops as a result of irreversible pancreatic β-cell destruction leading to degranulation and reduced insulin secretion [30]. STZ-induced diabetes was characterized by a severe loss in body weight, which has also been reported by other researchers [31]. The decrease in body weight in diabetic rats may indicate loss or degradation of structural proteins, which have been reported to contribute to body weight [32]. The present study showed that glibenclamide and metformin did not prevent weight loss. However, administration of honey alone or in combination with glibenclamide and metformin significantly improved body weight. Since glibenclamide and metformin, despite their hypoglycemic effect, did not have any effect on body weight, this shows that improvement of body weight by tualang honey is probably not related to its hypoglycemic effect but perhaps, it is acting through other mechanism(s).

## 5. Conclusions

This study shows that oxidative stress is still evident in the pancreas of diabetic rats administered the two most commonly prescribed antidiabetic drugs, glibenclamide and metformin. The administration of these two drugs in combination with tualang honey inhibited lipid peroxidation and attenuated the altered activities of antioxidant enzymes. In other words, based on our data, it can be said that tualang honey provides additional antioxidant effect to glibenclamide and metformin. Thus, the additional antioxidant protection exhibited by a combination of glibenclamide, metformin and tualang honey can be ascribed to tualang honey. This protection on pancreas against oxidative stress might also partially have contributed to the hypoglycemic effect of tualang honey in diabetic rats. This study establishes a basis for the need of antioxidant therapy in combination with hypoglycemic drugs in the management of diabetes mellitus.

## Figures and Tables

**Table 1.** Rat treatment groups.

**Group**	**Treatment**
1	(Normal) Distilled water (0.5 mL)
2	(Normal) Tualang honey (1.0 g/kg/body weight)
3	(Diabetic) Distilled water (0.5 mL)
4	(Diabetic) Tualang honey (1.0 g/kg/body weight)
5	(Diabetic) Glibenclamide (0.6 mg/kg/body weight) + metformin (100 mg/kg/body weight)
6	(Diabetic) Glibenclamide (0.6 mg/kg/body weight) + metformin (100 mg/kg/body weight) + tualang honey (1.0 g/kg/body weight)
